# Permanent His-bundle pacing in the right atrium in a patient with a Mobitz II atrioventricular block: a case presentation

**DOI:** 10.1186/s13019-021-01747-w

**Published:** 2022-01-08

**Authors:** Zai-Qiang Zhang, Jia-Wang Ding

**Affiliations:** 1 Department of Cardiology, The First College of Clinical Medical Sciences, China Three Gorges University, 183 Yiling Road, Yichang, 443000 Hubei People’s Republic of China; 2 Institute of Cardiovascular Diseases, China Three Gorges University, Yichang, 443000 Hubei People’s Republic of China

**Keywords:** His-bundle pacing, Mobitz II atrioventricular block, Pacemaker, Case report

## Abstract

**Background:**

This case report presents a patient diagnosed with sick sinus syndrome who was successfully treated with permanent His-bundle pacing (PHBP).

**Case presentation:**

A 36-year-old man was transferred to our hospital due to recurrent syncope. He was diagnosed with sick sinus syndrome based on the 24-h Holter and a history of syncope. He was admitted to hospital and successfully treated with PHBP. The postoperative examination showed that the pacing rhythm, pacemaker pacing and perception function were normal. He was discharged without any complications after a successful pacemaker implantation.

**Conclusions:**

We described a case in which PHBP may become an optimal approach to the management of patients with sick sinus syndrome. Right ventricular pacing has been attempted with inconsistent efficacy outcomes. HBP provides a promising alternative pacing option that might provide symptom resolution to patients with sick sinus syndrome.

## Introduction

At present, cardiac pacing is one of the most effective treatments for sick sinus syndrome. The right ventricular apex is the classical pacing site and has the characteristics of convenient electrode placement and a stable threshold. Recent studies have shown that significant long-term right ventricular apical pacing is similar to a left bundle branch block, which can result in dyssynchronous contraction with delayed lateral left ventricular motion and an increase in the incidence of atrial fibrillation (AF), hospitalization rate for heart failure and all-cause mortality [1–3]. As it provides the most physiological form of ventricular activation when compared to other pacing modalities, permanent His-bundle pacing (PHBP) has emerged as a new, promising approach to deliver physiological pacing, maintaining long-term ventricular synchrony and left ventricular performance, unlike conventional right ventricular apical pacing, for the treatment of patients with symptomatic bradycardia [4]. We present an interesting case of a 36-year-old man with sick sinus syndrome who was successfully treated with PHBP.

## Case presentation

**Fig. 1:**
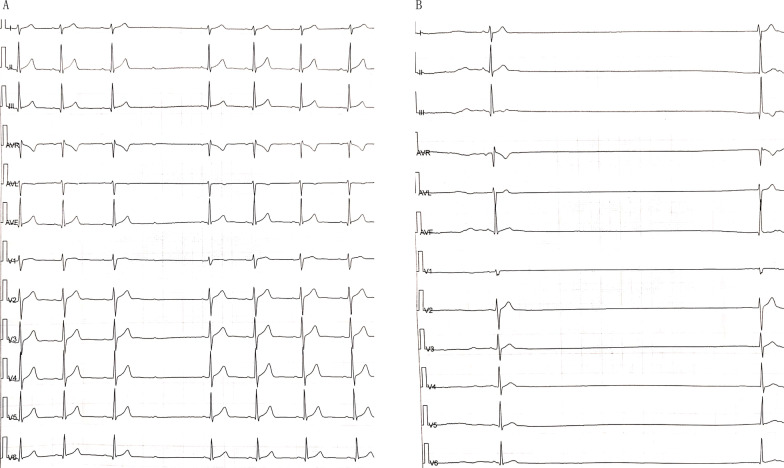
**a** The 24-h Holter exhibited sinus rhythm, Mobitz II atrioventricular block. **b** The 24-h Holter exhibited sinus arrest with the longest time being 5148 ms

**Fig. 2:**
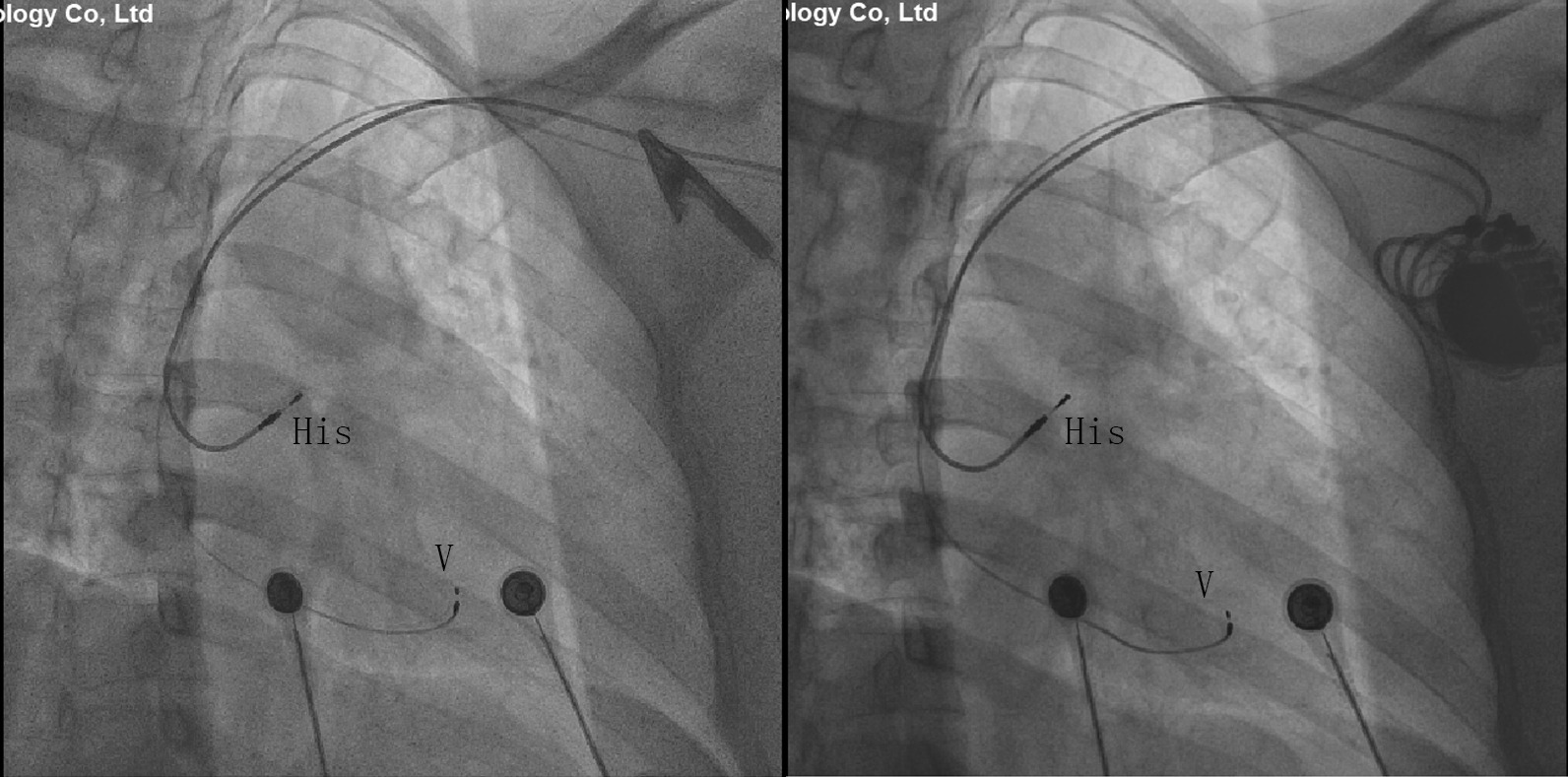
Successful implantation of His-bundle pacemaker in a cardiac catheterization laboratory

**Fig. 3:**
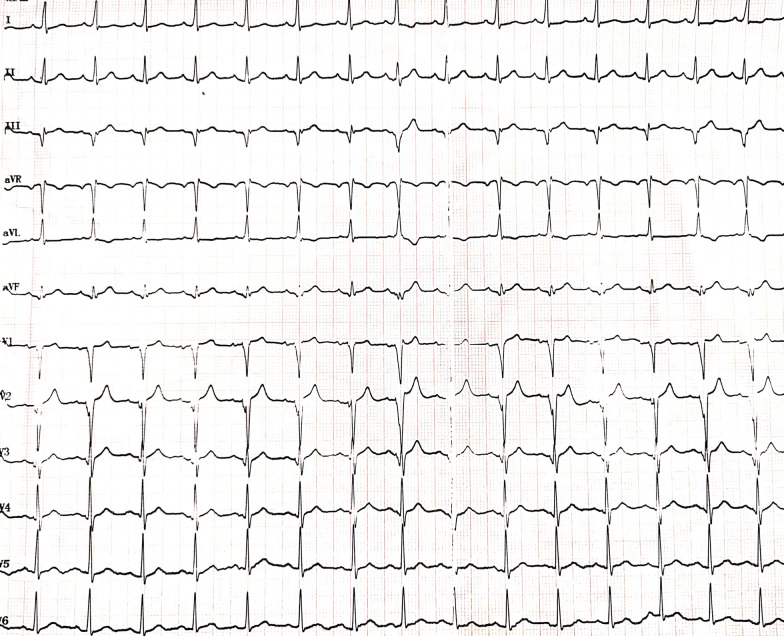
The postoperative electrocardiogram (ECG) showed sinus rhythm, a dual chamber pacemaker, and DDD mode pacing, and pacemaker pacing perception function was normal

A 36-year-old man was transferred to our hospital due to recurrent syncope. His symptoms started approximately 1 month prior to presentation. He had no past medical history. The 24-h Holter on admission exhibited sinus rhythm, a Mobitz II atrioventricular block and sinus arrest, with the longest time being 5148 ms (Fig. 1). Transthoracic echocardiography revealed normal ventricular function without any significant abnormalities. Cardiac CTA showed no significant coronary lesions. After completing the relevant inspection, he underwent permanent dual chamber pacemaker (Advisa DR, Medtronic Inc., Minneapolis, MN, USA) implantation (Fig. 2). The postoperative electrocardiogram (ECG) showed sinus rhythm, a dual chamber pacemaker, and DDD mode pacing, and pacemaker pacing perception function was normal (Fig. 3).

The pacemaker implantation procedure was performed in a cardiac catheterization laboratory. Under local anesthesia, the left subclavian vein was punctured, and a C315 His sheath (Medtronic, Minneapolis, MN) was advanced into the right atrium (RA) over a guidewire. Thereafter, the 3830 His lead (Medtronic, Minneapolis, MN) was advanced to the tip of the sheath. The parameters of the electrode were as follows: ventricular threshold: 0.4 V, perception: 12.0 mV, impedance: 650 Ω. The pacing electrode (Medtronic, 6232ADJ) was fixed after the incision. The detachable sheath was sent along the guidewire, and the atrial pacing electrode was sent to the right atrial auricle through the detachable sheath. The electrode position was adjusted, and the following electrode parameters were tested: ventricular threshold: 0.5 V, perception: 3.0 mv, impedance: 500 Ω. The pacing electrodes were fixed. The pacemaker was connected and implanted into the premade pacemaker bag (this was a pacemaker bag made in advance). The postoperative examination showed that the pacing rhythm, pacemaker pacing and perception function were normal. He was discharged without any complications after a successful pacemaker implantation (pacing mode: DDD 55–130 bpm, sense AV delay: 80 ms, pace AV delay: 100 ms, RA sensing 0.3 mV + auto capture managing on, RV sensing 0.9 mV + auto capture managing off, RA output 2.5 V × 0.4 ms, RV output 4.0 V × 1.0 ms).

## Discussion

Traditional right ventricular apical pacing can lead to inhomogeneous ventricular hypertrophy, abnormal diastolic function, cardiac rehearsal disorder and fibrosis, ventricular asynchrony and left ventricular structural change [5]. In addition, there are still many deficiencies in dual ventricular resynchronization pacing. HBP, a landmark breakthrough in pacing technology in recent years, is a potential physiological pacing mode for preventing and treating heart failure. A number of studies have shown that His-bundle pacing is close to physiological pacing, which can reverse ventricular remodeling, restore ventricular electrical and mechanical synchronization, and improve cardiac function [6]. At present, there are small samples of studies that suggest that HBP can alleviate the symptoms of heart failure and enhance ventricular pumping function in some people, but large samples in randomized clinical studies are still needed to clarify the safety and clinical benefits of HBP in clinical practice.

We present a case of a patient who underwent implantation of a permanent dual chamber pacemaker for symptomatic sick sinus syndrome. Patients with a markedly prolonged PR interval can have decreased exercise tolerance owing to pseudo-pacemaker syndrome [7]. A dual chamber pacemaker has been traditionally implanted in these patients to improve AV synchrony, with subsequent improvement in exercise tolerance. Given the deleterious effect of RV pacing [8], HBP provides physiological activation of both ventricles by recruiting the conduction system, which has obvious advantages.

In this case, we map the His region through the Pacing Systems Analyzer.According to the ECG after his bundle pumping, It can be divided into two categories: (1) selective His bundle pacing (S-HBP), and its cardiac electrophysiological standard is: ① the shape and time course of pacing QRS wave on body surface ECG are the same as its own rhythm; ② PV interval after pacing signal is basically equal to HV interval. (2) non selective his bundle pacing (NS-HBP), the common criteria are: ① low output pacing activates the endocardial myocardial tissue near his bundle, and the QRS wave is wide, while high pacing output activates the conduction pathway and narrows the QRS; ② His bundle potential can be recorded on the pacing lead; ③ PV interval < HV interval; ④ QRS wave generated by pacing is consistent with its own P-wave axis; ⑤ QRS wave generated by pacing is on the body surface The duration of ECG was at least 50 ms less than that of right ventricular apical pacing.

The threshold of HBP is higher than that of conventional implantation site, which is prone to microdislocation and local tissue fibrosis, resulting in postoperative threshold increase and loss of capture [9]. Compared with normal right ventricular pacing, the threshold of HBP lead is higher and the amplitude of R wave is lower. The possible reason is that HBP mostly senses ventricular far-field potential, and the amplitude of his bundle lead is much smaller than ventricular potential; the higher threshold may be related to less myocardial tissue and rich fibrous tissue at the implantation site.

This case provides several interesting teaching points. First, patients with a prolonged PR interval and sinus arrest should be carefully evaluated for the cause of their symptoms. Second, for young patients without obvious cardiac insufficiency, HBP should be the preferred mode of pacing, as it not only restores AV synchrony like dual-chamber pacing but also maintains interventricular synchrony, which may further improve cardiac function and prevent pacing-induced cardiomyopathy. Third, the success rate of HBP implantation is approximately 85%. Although the success rate of HBP implantation has improved with the development of special devices, it also decreases the learning difficulty. His-bundle pacing is technically challenging to perform and requires precise mapping of the His bundle. Although His-bundle pacing is more physiological than right ventricular pacing, most of the current clinical studies are single-center retrospective studies. More large sample, multicenter, prospective randomized controlled studies are still needed to evaluate the safety and effectiveness of His bundle pacing.

## Conclusion

We consider that in young patients (such as the one presented here), HBP is an alternative worth considering. However, the limitations of permanent His-bundle pacing are the inability to implant the lead on the His-bundle in 10–20% of the patients and the lack of available randomized large-scale data to justify the use of His-bundle pacing in all cases.

## Data Availability

The datasets of the current study are available from the corresponding author upon reasonable request.

## Abbreviations

AF: Atrial fibrillation
PHBP: Permanent His-bundle pacing
ECG: Electrocardiogram
RA: Right atrium
